# Preoperatory Anxiety in Patients Undergoing Cardiac Surgery

**DOI:** 10.3390/diseases7020046

**Published:** 2019-06-19

**Authors:** José Prado-Olivares, Elena Chover-Sierra

**Affiliations:** 1 Internal Medicine Unit. Hospital Universitario de Mostoles, 28935 Madrid, Spain; josecarlosprado7@gmail.com; 2 Nursing Department, Facultat d’infermeria i podologia, 46001 Valencia, Spain; 3 Internal Medicine Unit. Hospital General Universitario, 46014 Valencia, Spain

**Keywords:** preoperative anxiety, cardiac surgery, STAI-S, patient’s education

## Abstract

Anxiety is a feeling of discomfort produced in face of an unknown event, as an impending cardiac surgery, that can lead to inconveniences in the intervention and subsequent recovery. Being the purpose of this research to analyze pre-surgical anxiety, a descriptive cross-sectional study among patients undergoing cardiac surgery was carried out. Data about sociodemographic variables were collected and the level of anxiety prior to surgery was assessed using the STAI-S scale. Subsequently, descriptive data analyses were performed, relationships among variables were analyzed, and a binary logistic regression model was developed in order to analyze the role of the variables involved in the development of preoperative anxiety. Sixty subjects were finally included; more than 80% had a moderate to high level of anxiety. 26.7% underwent valve surgery and 47% underwent coronary artery bypass graft (CABG) surgery, in the latter case presenting higher levels of anxiety. Statistically significant relationships were found among the level of anxiety and (a) level of studies, (b) first surgical intervention, and (c) the rating given to their previous surgical experience. We concluded that preoperative anxiety in people undergoing cardiac surgery is high and yet it is an underestimated phenomenon. The relationship between the received information and their anxiety level is inversely proportional, so that people programmed for cardiac surgery should be provided with all the information they required, through an individualized intervention.

## 1. Introduction

Anxiety is defined as an emotion or feeling of non-specific discomfort that occurs in the face of an event unknown to the individual. It is also a complex reaction that occurs in the face of events subjectively perceived as dangerous, although it is due solely to the fact of being uncertain [1].

According to Spielberg, this can be subdivided into trait anxiety, as a stable feature of personality, and state anxiety, as the degree of anxiety at a particular time [2]. The latter is the type of anxiety analyzed in this study, since we are interested in knowing the level of anxiety at the moment prior to cardiac surgery.

Any surgery generates a level of anxiety, because the patient perceives the act as dangerous [1], however, the fact of being a candidate for cardiac surgery would raise the level of anxiety even more, since this type of interventions, such as coronary artery bypass graft (CABG) and valve replacement surgery, are related to a high vital risk [3,4,5].

There are copious studies that indicate that a high level of preoperative anxiety negatively affects the intervention itself and the subsequent recovery. According to Osco Torres et al. and Teixera-Lima et al. [3,6], when a person has a high degree of anxiety, this can influence the body’s response to anesthetic drugs and an increase in heart rate and blood pressure. If we consider that we are talking about cardiac surgery, the modification of these parameters could suppose an added risk that must be taken into account.

Regarding the postoperative period, Carapia Sadurni et al. [7] indicated that the higher level of pre-surgical anxiety, the longer and more difficult the convalescence became. Similarly, Moix Queraltó [8] states that this anxiety can affect different indicators of recovery such as pain, analgesics and sedatives needing, nausea, and changes in body temperature and blood pressure.

Due to these drawbacks, it is necessary to evaluate and prevent anxiety prior to surgery, since this way it is assumed that it will increase patient satisfaction, and as Osco Torres et al. and Díez Álvarez et al. affirmed, possible intraoperative complications related to the intervention and anesthesia would be reduced [6,9]. The time of postoperative stay would also be shortened, assuming a lower economic cost for the hospital, as well as a reduction in waiting lists.

In the specific context of cardiac surgery, which supposes a high vital risk in many cases, different research studies have been carried out [4,5,10,11,12,13]. Their purpose was to evaluate the levels of preoperative anxiety present in the patients and also to identify which were the factors related to patient and surgery characteristics, as both could influence the development of preoperative anxiety.

Thus, high levels of anxiety have been found prior to cardiac surgery to depend on different variables such as patient’s age and gender, level of studies, previous surgical experiences, and so on. These higher levels of anxiety have been related in many cases to postoperative pain, even at long term, as it was found by Choinière et al [14], who analyzed nonanginal pain until two years after cardiac surgery in a big cohort of people submitted to this surgery, finding that anxiety levels could be predictor of this postsurgical nonanginal chest pain.

For this reason, we consider as the objective of our work to evaluate preoperative anxiety in patients who were undergoing cardiac surgery in the next few h, and to analyze the factors that influence the development/presence of moderate anxiety levels. The purpose is to define the profile of patients who would have higher levels of anxiety prior to cardiac surgery and who may require greater need for prior intervention to control their anxiety.

## 2. Methods

### 2.1. Study Design

A descriptive, analytical, cross-sectional study was carried out at a tertiary Hospital in Valencia (Spain) during a period of 2 months (March and April 2015), in patients who were hospitalized the day before a cardiac surgery, in order to assess anxiety prior to surgical procedure depending on certain variables.

### 2.2. Study Participants

All those patients scheduled to undergo cardiac surgery in the following 24 h, in a Spanish hospital, between 3 March and 4 May, 2015, form the sample used for this study. Due to the kind of study, the subjects were selected by convenience sampling, including those subjects who met the inclusion criteria (patients scheduled for cardiac surgery the following day, over 18 years of age, who had signed informed consent). Those subjects with psychological/psychiatric alterations and/or with difficulties in communication or in understanding the questionnaires were excluded.

### 2.3. Data Collection

A data collection document was elaborated, with some questions about sociodemographic features and the participants’ type of surgery (independent variables) as well as the STAI-S scale to know the level of anxiety-state (dependent variable).

#### STAI

Several instruments have been used, such as the visual anxiety scale or the HADS, in the multiple studies that have been carried out around the world to assess the level of presurgical anxiety. This variety of instruments is shown in these two studies by Guo [11] and Tavares-Gomes [15], in which the different instruments used for this purpose are reviewed. In our case, it has been decided to use the STAI scale, specifically the subscale of state anxiety (STAI-S), as it is one of the most widely used in researches performed in Spain or in Spanish and Portuguese speaking countries, and because of its ease of use and little time needed to complete it. In addition, some works consider the STAI as the gold standard in anxiety assessment [16].

STAI is a scale elaborated by Spielberger, Gorsuch, and Lushene in 1970 [2], for assessing the anxiety based on the model of the Spielberger, where the anxiety is divided into two factors: Anxiety as a personality trait, and anxiety as a situational state.

It has been translated into more than 40 languages and has proven to be useful in the assessment of anxiety in several contexts. Regarding its psychometric properties, it has shown a satisfactory internal consistency (Cronbach’s alpha 0.86–0.95) and optimal results in terms of test-retest reliability. It was adapted to Spanish some years ago, providing reliability data similar to the original version, and it is a reference when evaluating anxiety [17,18].

As for its structure, it consists of two scales with 20 items each one, formulated alternately in positive and negative. Since each scale measures different aspects, the instrument allows applying only the scale that best suits the investigation. In this case, the STAI-S scale (anxiety - state) was used, because it assesses the level of anxiety of the subject at a specific moment, where the patient must answer the questions the way he feels at that moment, on a Likert-type scale (1—“nothing”, 2—“a little”, 3—“quite”, and 4—“a lot”) [2,18].

It allows researchers to obtain a numerical value that represents the level of anxiety and also establishes categories in this level of anxiety, when classified as “High Anxiety” if the score is greater than or equal to 45, “Average Anxiety” when the score ranges between 30 and 44 points, and “Mild anxiety” if the score is less than or equal to 30, as indicated in the rating strategy of the scale validation [18,19]

### 2.4. Ethical and Administrative Considerations

The project was approved by the Research and Ethics Committee of the hospital. An “informative document” was prepared to explain the purpose, risks, and benefits of participating in the study. This so-called “informed consent” that had to be signed was attached to it and the confidentiality of the collected data was reported.

### 2.5. Statistical Data Analysis

The SPSS^®^ v.21 software (Armonk, NY: IBM Corp. Armonk, New York, NY, USA) for Microsoft Windows© was used to perform the statistical analysis. Firstly, the descriptive univariate analyses were performed. For those quantitative variables, central tendency measures and measures of dispersion were studied. In the case of qualitative variables, frequency distribution was analyzed. Secondly, correlations among numerical variables were analyzed using Pearson’s parametric test. Next, means difference tests among two or more groups were performed, using non-parametric tests due to the size of the sample. In all the analyses a confidence level of 95% was established (*p* < 0.05)

A binary logistic regression model was also developed with the aim of being able to identify predictors of results (anxiety level). In this binary logistic regression model, the independent variable became dichotomous (1 = moderate and high anxiety/0 = low anxiety). Categorical and numerical variables were used as explanatory ones (ordinal variables such as level of education and assessment if their previous surgical experience were considered as numerical ones). Then a guided backward procedure of comparisons of hierarchical models was carried out in order to select the best-fitted. The process was based on dropping in a sequential way (one each time) those variables and interactions whose effects had higher and non-significant *p*-values in the Wald test.

## 3. Results

### 3.1. Features of Participants

60 people, with an average age of 63 years, participated in the study. Other population’s characteristics are shown in Table 1.

Regarding the type of surgery to which they were going to undergo, in 25 cases it was a CABG surgery and 16 were scheduled for valve replacement, while 31.7% of subjects underwent both types of surgery.

These patients were asked about their satisfaction and 72.1% indicated that they had a good or very good experience with surgery, while the remaining 27.9% rated their previous surgical experience as bad or very bad.

It also draws attention that, although most of the participants reported having received some information in the preoperative period, more than half of the subjects (53.3%) manifested their need for more information about the surgical, anesthetic, and postsurgical recovery procedure.

### 3.2. Anxiety Levels Analysis

After analyzing the results of the STAI-S scale, we observed that the participants presented an average score of 40 points (40.44 ± 11.93; Range: 22–67), which would correspond to a level of moderate anxiety. If we analyzed these values as a category we would see that 43.3% of the subjects presented a "high anxiety" (>45 points), 40% (30–45 points) have “moderate anxiety”, and only 16.7% presented a "low anxiety" (<30 points).

### 3.3. Relationships between Anxiety Levels and Features of the Studied Population

**Figure 1:**
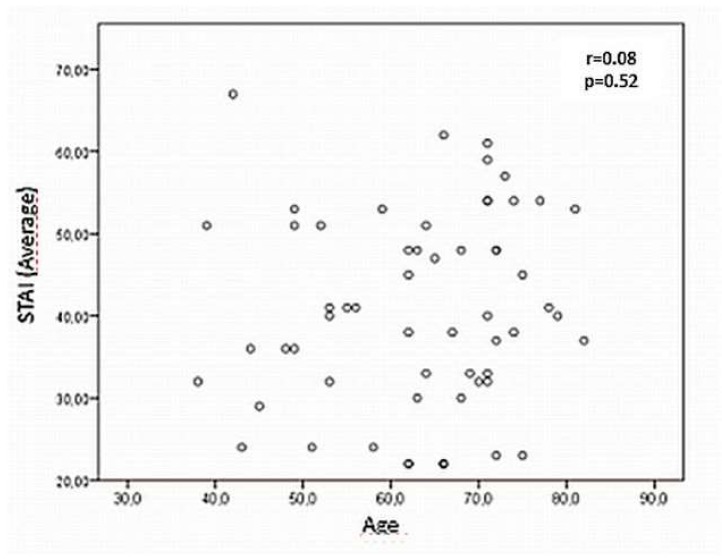
Correlation between anxiety scores and participants’ age.

Figure 1 shows the correlation between the levels of anxiety referred by the participants and their age, a relationship that was not statistically significant, although it is striking that the older they were, the higher their level of anxiety was.

Table 2 presents the results of the anxiety score based on the descriptive characteristics of the population, in which we see the existence of statistically significant differences in the results according to variables such as their level of education and the assessment of their previous surgical experiences.

It is also necessary to add that people who reported needing more information about their surgery also had higher anxiety scores (43.44 ± 11.85 compared to 39.21 ± 11.31 in the group of those who reported not needing more information), although these differences were not statistically significant.

### 3.4. Bivariate Linear Model, Explanatory of the Factors which Influence the Preoperative Anxiety in the Studied Population

Table 3 shows the coefficients of the binary logistic regression model and the odds ratio associated with the variables included in the model.

Thus, it can be appreciated that people who have undergone previously some type of surgery and who evaluate more positively their previous surgery, the youngest ones, and also those with higher levels of education, will be less likely to develop moderate or high levels of anxiety.

## 4. Discussion

Pre-surgical anxiety in people undergoing cardiac surgery is a common phenomenon, since the fact of being operated on for such a complex surgery generates insecurity in the patient, and this is demonstrated by the results, which indicate that more than 80% of the sample studied presented an important level of anxiety. These data are similar to those found in previous studies such as that of Valenzuela Millán [20], where 76% of the subjects showed high anxiety, as well as the study by Castillero Amador [10] about psychological intervention in cardiac surgery, where 98% of participants presented anxiety, and 48% of them did so in an elevated way. This result is consistent with the percentage of patients who showed high anxiety in the present study, which was 43.3%.

Although the state of mind carries important repercussions during the intervention and postoperation, it is sometimes underestimated. In this sense, according to Carapia Sadurni [7], preoperative anxiety is associated with a greater frequency of anesthetic accidents, greater vulnerability to infections, longer hospital stay, and higher levels of postoperative pain.

In addition, Castillero asserts in [10] that patients with a higher level of anxiety are those who complain the most and who have greater difficulty in following instructions from the health personnel such as coughing, breathing deeply, mobilizing or feeding, so that the recovery period in hospital would lengthen accordingly. It even indicates that high levels of anxiety can alter neuroendocrine homeostasis, producing a deficit of secretion in growth hormone, delaying tissue repair (healing).

Regarding sociodemographic variables, a statistically significant, inversely proportional relationship (*p* < 0.05) was found between the level of studies and the level of anxiety. These results are similar to those obtained by Diez Álvarez et al., who found that patients with university studies tend to have a lower degree of anxiety [9].

It is likely that the higher the level of education, the greater the capacity to understand the information offered, in the same way that those people who have already had a previous surgical experience know what they will face in terms of the anesthetic process and later recovery. Considering that anxiety is defined as fear of the unknown, it could be postulated that the quantity/quality of information about the surgical process contributes to the reduction of presurgical anxiety, as it was considered by Ruiz-López et al. [21] and Fahti et al. [4] as well.

Another data that could reinforce the previous idea is that, despite not presenting a statistically significant relationship (*p* = 0.105), we can observe a clear relationship between the level of anxiety and the need to obtain more information regarding the intervention: People who expressed this desire (53.3%) presented levels of anxiety higher than the group that was satisfied with the information provided. This hypothesis is supported by studies such as the one carried out by Kiyohara et al. [22] and the one performed in Spain by Doñate et al. [23] where a statistically significant and inverse relation was found between the amount of information and the preoperative anxiety. It must be clarified that the information that most managed to reduce anxiety was that referring to the surgical procedure and not so much to the diagnosis or anesthetic process. This is a very striking fact in the population that participated in this study, since all of them were in patients to undergo scheduled (not urgent) surgery and who had had a pre-anesthetic/pre-surgical visit in which they had received information about the process. Thus, this study, as do others, shows the importance of improving the information provided to the patient, as well as improving the communication skills of health professionals.

In this line, other studies have been carried out, such as the one conducted by Galego Vázquez and Peña Valiño [24], where 56% of the patients intervened considered that the presurgical information was insufficient. According to another study by León Castro and Salazar Vargas, less than 50% of patients scheduled for cardiac surgery were able to describe the intervention, and less than 33% knew how the postoperative course would be like.

If, in addition, it is taken into account that in the present study a statistically significant relationship has been established (*p* < 0.05) between the qualification of the previous experience (in those cases that occurred) and the level of anxiety directly, it reaffirms the idea that the information that is possessed, in the form of previous experience, influences pre-surgical anxiety. In the studies developed by Gallego Vázquez and Peña Valiño [24], this relationship is also supported, since they affirm that a bad experience negatively influenced pre-surgical anxiety in the face of a new intervention. Bages Fortacin [18] et al. also studied this effect of previous surgical experience in terms of the level of anxiety that patients presented, and it was found that those patients with previous positive experiences had lower levels of preoperative anxiety, as well as those who considered the information that they had received about their process was sufficient. 

Therefore, it is understood that a greater quantity and quality of information would reduce anxiety levels. According to Gordillo et al. [25], although some studies could point out that more information would raise the level of anxiety, the fact is that 82.3% of the subjects reported not having a subjective feeling of anxiety when receiving said information, and 87.7% indicated that they preferred to know what diagnosis they had and also the proposed intervention. In the same study, 91.3% of the subjects obtained high anxiety in STAI-S did not receive prior information, while 93.3% of the subjects who obtained normal or low anxiety, knew what the proposed intervention consisted in. For this reason, several works such as Wongkietkachorn et al. [20] have tried to adjust the information provided to each patient to their own needs. Thanks to it, better results in the control of postoperative anxiety have been obtained in the group that had received the information as requested by them than in the group that received standardized information.

Likewise, people who were facing a surgical intervention for the first time showed a higher level of anxiety (*p* < 0.01) than those who had already undergone another intervention. Such lower level of anxiety in patients who already had previous experience with surgery was also identified in Quintero’s and Matthias’ researches [16,26]. On the other hand, Negromonte-Gonçalves et al. [13] also found statistically significant differences among anxiety levels in those persons participating in their study who underwent a previous cardiac surgery, the group with higher anxiety levels.

Regarding the type of intervention, those participants proposed for coronary artery bypass intervention (coronary bypass) presented a higher level of anxiety than those whose intervention would be valvular surgery (valve replacement or valvular plasty), although the latter is more complex and requires the use of extracorporeal circulation on all occasions. This phenomenon could be explained by the fact that those people undergoing coronary revascularization surgery have been previously diagnosed with a coronary problem (angina pectoris or acute myocardial infarction), which already generates a high level of anxiety. Meanwhile, valvulopathies present a more silent and nonspecific symptomatology and therefore are less likely to be associated with imminent life risk, not raising the level of anxiety. Something important to analyze in future work would be to assess the level of anxiety presented by patients who are going to be subjected to new techniques of minimally invasive cardiac surgery, such as TAVI surgery for valve replacement. In a study carried out in our country by Hernández-Palazón et al. [5], and in the one developed in Sri Lanka by Matthias [16] this higher level of anxiety was also identified in people undergoing CABG, so this should be an aspect to consider. It should be also taken into account the fact if it would be convenient to explain to the patient the type of intervention in detail. 

Despite not having found statistically significant relationships, we highlight that the fact of being a woman would suppose a higher level of anxiety, as well as the result obtained in Spain by Diez-Alvarez [9] and in Valenzuela Millán’s study [20], where 70% of the subjects with high anxiety were female, and also in a study among people scheduled for cardiac surgery developed in Iran [4]. This relationship between gender and anxiety is also found in a research performed in Brazil by Rodrigues et al. [27], using HADS to assess presurgical anxiety; in the study performed by Negromonte-Gonçalves et al. in which the instrument used to assess anxiety levels was the Beck anxiety inventory [13]; and also in a study developed in Sri Lanka using the APAIS scale as an anxiety measurement instrument [16]

Regarding age, despite not having been able in this study to establish that it is statistically significant, it has been possible to observe the existence of a direct relationship with the STAI-S score. This relationship between patients’ age and the development of presurgical anxiety has been also analyzed in other studies, without a homogeneity in these results [4,13,27].

The proposed logistic binary regression model shows that the aspects related to the participants’ personal experiences with previous surgery, together with the level of studies of the person, are both variables which will have the most influence in the non-development of presurgical anxiety. Although it is true that each of them has a different weight in this protective role, being the level of studies the one that would confer greater protection against the development of this anxiety.

Studies such as those of Orihuela Perez, Sjöling et al. and Carrascosa López et al. [28,29,30] indicate that a presurgical visit would favorably affect the reduction of anxiety-state level, facilitating the coping of surgery, while Díez Álvarez [9] specifies that the immediate preoperative (1 h before the intervention) would not be the ideal time for an approach to anxiety, since the anxiolytic effect of the intervention is very low with so little anteriority. On the other hand, studies such as that of Ortiz et al. [31] showed that an educational intervention improved patients’ knowledge about the surgical intervention, but did not reduce the levels of anxiety referred to by them.

Therefore, it would be convenient to design an intervention strategy and a pre-post study to evaluate its effectiveness, as it has been done in several studies that analyze the effectiveness of interventions aimed at reducing the anxiety referred by patients [9,11,30,32]. However, it must be considered that the information to be provided to patients should be personalized, depending on their level of knowledge and their needs for more information, and that it is interesting to explore previous experiences related to surgery in presurgical assessment. This need to adapt the offered information to the characteristics of each subject to reduce their levels of anxiety is something that other studies also talk about, indicating that this adaptation could have a greater effect in reducing these levels of presurgical anxiety. In several of these works, nurses are the health professionals responsible for offering the information or carrying out the educational activity, which is a sample of the important role of nurses in this process [9,13,32].

The main limitation for the realization of the present study is related to the sample size and the non-randomized selection of the participants in the study, which makes it difficult to generalize the results. Even though, we have been able to detect significant differences between the two principal types of surgery and we have identified in the regression model some important factors that modulate anxiety levels which are the experience with previous surgical processes and the subjects’ educational level (not reported before).

Another limitation is that the previous anxiety state of each patient was not considered; and even not having used, for instance, the trait subscale of STAI, to identify those subjects who already had high baseline anxiety. Although we excluded from the study people with psychiatric/psychological pathologies that could present high levels of anxiety in order to try to control these previous levels of anxiety, preexisting anxiety has not been evaluated yet, and therefore we may have overestimated the anxiety levels of our patients.

## 5. Conclusions

After analyzing the results obtained in the present study, and comparing them with other studies related to presurgical anxiety, it could be concluded that a large majority of people, faced with an impending cardiac surgery intervention, have a high level of anxiety. This fact would be related to the education level of the subjects, as well as having had prior surgical experiences, and satisfaction with them.

In many cases, patients feel that they have not received enough information about the surgery they are undergoing, and this fact can cause an increase in their anxiety levels. It would be convenient to assess the anxiety and reduce it as much as possible, providing the corresponding information in a comprehensible and individualized manner and allowing at any moment to express doubts about the diagnosis, intervention, and recovery of the surgery. All this using a clear language, leaving aside the sanitary jargon, adapted to the level of the user.

## Figures and Tables

**Table 1:** Features of studied population.

	N	(%)
**Gender**		
Female	35	58.3%
Male	25	41.7%
**Marital status**		
Single	4	6.7%
In union	4	6.7%
Married	35	58.3%
Separated	3	5.0%
Divorced	2	3.3%
Widow/er	12	20.0%
**Level of education**		
No studies	7	11.7%
Primary studies	30	50.0%
Secondary studies	15	25.0%
University studies	8	13.3%

**Table 2:** Anxiety scores according to descriptive features of the studied population.

	STAI-S Score (Mean ± SD)	*p*-Value
**Gender**		*p* = 0.13
Men	38.92 ± 13.94	
Women	43.29 ± 9.59	
**Level of education**		* *p* < 0.05
No studies	52.57 ± 8.66	
Primary studies	40.9 0± 11.46	
Secondary studies	36.26 ± 10.68	
University studies	36.13 ± 10.68	
**Surgery type**		*p* = 0.86
CABG	42.60 ± 13.86	
Valvular	40.8 1± 10.94	
Both	40.53 ± 9.45	
**Previous surgery**		** *p* < 0.01
Yes (n = 43)	48.50 ± 9.06	
No (n = 17)	37.44 ± 11.56	
**Previous surgery’s assessment**		* *p* < 0.05
Very good (n = 13)	32.07 ± 7.94	
Good (n = 18)	33.83 ± 11.20	
Bad (n = 11)	47.73 ± 6.98	
Very bad (n = 1)	59.00	

* Kruskal–Wallis test; ** Mann–Whitney test.

**Table 3:** Coefficients of the binary logistic regression model.

	β	*p*-Value	OR	OR CI (95%)
Age	−0.14	<0.05	0.87	0.76–0.99
**Previous surgery’s assessment**	−2.29	<0.05	0.10	0.02–0.57
**Level of education**	−3.11	<0.01	0.04	0.01–0.38
